# Hypersensitivity Induced by Intrathecal Bradykinin Administration Is Enhanced by N-oleoyldopamine (OLDA) and Prevented by TRPV1 Antagonist

**DOI:** 10.3390/ijms22073712

**Published:** 2021-04-02

**Authors:** Eva Uchytilova, Diana Spicarova, Jiri Palecek

**Affiliations:** 1 Laboratory of Pain Research, Institute of Physiology, Czech Academy of Sciences, Videnska 1083, 14220 Prague, Czech Republic; eva.uchytilova@ikem.cz; 2 Department of Anaesthesiology, Resuscitation and Critical Care, Institute for Clinical and Experimental Medicine, Videnska 1958/9, 14021 Prague, Czech Republic

**Keywords:** TRPV1, bradykinin, OLDA, spinal cord, hyperalgesia, allodynia

## Abstract

Transient receptor potential vanilloid 1 (TRPV1) channels contribute to the development of several chronic pain states and represent a possible therapeutic target in many painful disease treatment. Proinflammatory mediator bradykinin (BK) sensitizes TRPV1, whereas noxious peripheral stimulation increases BK level in the spinal cord. Here, we investigated the involvement of spinal TRPV1 in thermal and mechanical hypersensitivity, evoked by intrathecal (i.t.) administration of BK and an endogenous agonist of TRPV1, N-oleoyldopamine (OLDA), using behavioral tests and i.t. catheter implantation, and administration of BK-induced transient thermal and mechanical hyperalgesia and mechanical allodynia. All these hypersensitive states were enhanced by co-administration of a low dose of OLDA (0.42 µg i.t.), which was ineffective only under the control conditions. Intrathecal pretreatment with TRPV1 selective antagonist SB366791 prevented hypersensitivity induced by i.t. co-administration of BK and OLDA. Our results demonstrate that both thermal and mechanical hypersensitivity evoked by co-administration of BK and OLDA is mediated by the activation of spinal TRPV1 channels.

## 1. Introduction

Analgesic effect of transient receptor potential vanilloid 1 (TRPV1) agonists and antagonists was studied extensively and therapeutic potential in pain relief was demonstrated mainly in chronic neuropathic conditions [1,2,3,4,5,6]. Activation of the TRPV1 receptors localized in periphery is beneficial, following application of high-concentration capsaicin (TRPV1 agonist) patches that are widely used, especially in the treatment of postherpetic neuralgia, HIV-associated neuropathic pain, or musculoskeletal pain [7,8]. In comparison, spinal TRPV1 represent a possible therapeutic target [9], as they are involved in the development of several pathological pain states [5,10,11,12,13,14,15,16].

The TRPV1 is a polymodal, established noxious heat-gated cation channel that is also activated by several endogenous ligands and is sensitized by phosphorylation through intracellular cascades, triggered by activation of kinin and several other receptors [17,18]. In our previous studies, we demonstrated the sensitization of spinal TRPV1 to endogenous agonist N-oleoyldopamine (OLDA) through bradykinin (BK), tumor necrosis factor-alpha or peripheral inflammation [19,20]. Proinflammatory mediator bradykinin undergoes rapid degradation, the metabolite desArg^9^ BK acts preferentially on kinin B_1_ receptor (B_1_R), which is inducible during pathological conditions. Bradykinin preferentially activates B_2_R constitutively expressed in normal tissue [21]. It was suggested that constitutively expressed B_2_R underlie the acute effect of BK, and inducible B_1_R underlie the chronic effect [21,22].

Interestingly, peripheral nociceptive input increases the production of BK in the spinal cord [23] and direct intrathecal (i.t.) administration of BK or desArg^9^ BK induces hyperalgesia, whilst the B_1_R antagonist administration (i.t.) also attenuates Freund’s complete adjuvant-induced hyperalgesia [23,24]. Spinal BK-induced hyperalgesia was shown to be mediated by protein kinase A (PKA), protein kinase C (PKC), and extracellular signal-regulated kinase [25]. Further, the level of phosphatidylinositol-4,5-bisphosphate (PIP_2_) could play a regulatory role in the process of TRPV1 sensitization by BK [26,27,28]. In addition, it was demonstrated that subcutaneous capsaicin application up-regulated B_1_R mRNA and protein expression in spinal cord microglia that contributed to low doses of B_1_R agonist-induced thermal hyperalgesia [29]. The role of TRPV1 in thermal hyperalgesia induced by intraplantar injection of BK was shown using TRPV1-deficient mice [26]. Recently, the close interaction and reciprocal regulatory mechanism between B_1_R and TRPV1 on non-peptidergic fibers of primary sensory neurons and spinal astrocytes was suggested in neuropathic pain conditions [30].

The aim of this study was to examine the effect of spinal OLDA on thermal and mechanical hypersensitivity induced by i.t. administration of BK, and further investigate the role of spinal TRPV1 in the hypersensitivity induced by co-application of BK and OLDA.

## 2. Results

### 2.1. The Role of Spinal TRPV1 in the Thermal Hyperalgesia Induced by i.t. Bradykinin Treatment

**Figure 1:**
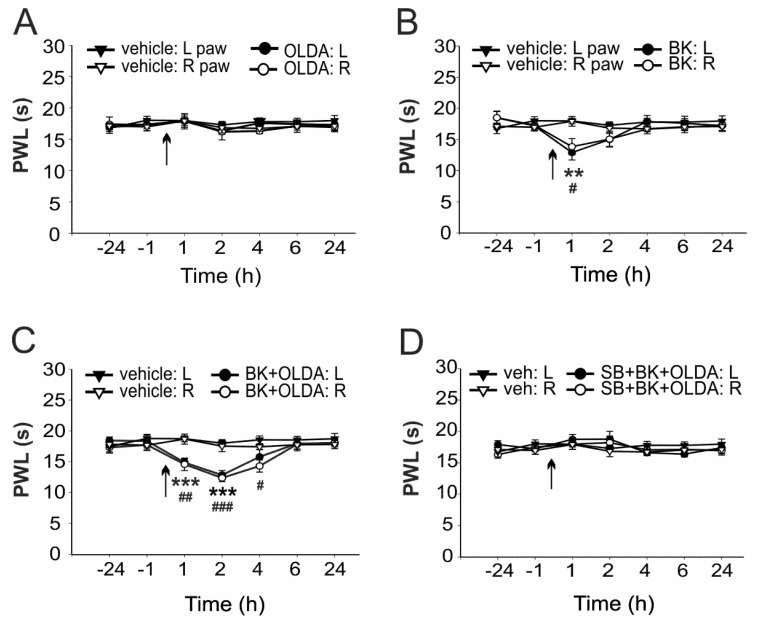
Bradykinin (BK)- and N-oleoyldopamine (OLDA)-induced thermal hyperalgesia was prevented by TRPV1 antagonist pretreatment. (**A**) Intrathecal (i.t.) administration of a relatively low dose of TRPV1 endogenous agonist OLDA (0.42 µg, *n* = 4) did not change the paw withdrawal latency (PWL), as compared to vehicle-treated rats (*n* = 6). (**B**) Administration of BK (21.2 μg, i.t., *n* = 6) transiently decreased the PWL, as compared to vehicle-treated rats. (**C**) Co-administration of both BK (21.2 μg) and OLDA (0.42 µg, i.t., *n* = 6) enhanced the hyperalgesic effect induced by BK alone. (**D**) Thermal hyperalgesia induced by BK and OLDA co-administration was prevented by selective TRPV1 antagonist SB366791 pretreatment (SB, 0.58 µg, i.t., 15 min, *n* = 7). Data represent the mean ± S.E.M. of 4–7 independent experiments. Statistical analysis: Mann-Whitney rank test; ** *p* < 0.01, *** *p* < 0.001 left hindpaw (L), BK versus vehicle treatment; ^#^ *p* < 0.05, ^##^ *p* < 0.01, ^###^ *p* < 0.001 right hindpaw (R), BK versus vehicle treatment. The time of the i.t. administration is marked by arrows.

**Figure 2:**
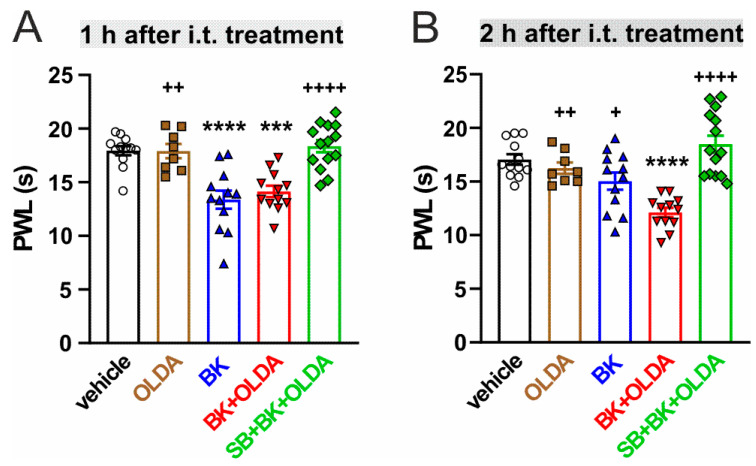
TRPV1 agonist i.t. treatment enhanced BK-induced thermal hyperalgesia in a time-dependent manner. A detailed comparison of the effects of the drugs, 1 h and 2 h after the i.t. administration. (**A**) Co-administration of BK (21.2 μg) and OLDA (0.42 µg) decreased the PWL 1 h after i.t. treatment to a similar level as BK alone. (**B**) Co-administration of BK and OLDA, enhanced the thermal hyperalgesia 2 h after i.t. treatment, as compared to BK alone. Data represent the mean ± S.E.M. with a scatterplot of the individual data points of 4–7 independent experiments. Statistical analysis: One-way ANOVA followed by Tukey’s test; *** *p* < 0.001, **** *p* < 0.0001, versus vehicle i.t. treatment; ^+^ *p* < 0.05, ^++^ *p* < 0.01, ^++++^ *p* < 0.0001, versus BK + OLDA i.t. treatment.

The contribution of spinal TRPV1 to the BK-induced thermal hyperalgesia was investigated using OLDA, an endogenous agonist of TRPV1, and selective antagonist SB366791. We used a relatively low dose of OLDA (0.42 µg), similar to our previous experiments that showed no effect on thermal hyperalgesia [20]. Intrathecal administration of OLDA (0.42 µg) did not significantly change the mean paw withdrawal latency (PWL) to radiant heat (*n* = 4, Figure 1A), as compared to pretreatment values. PWLs were also not changed by vehicle administration, as compared to control values before injection (*n* = 6). However, i.t. administration of BK (21.2 μg, *n* = 6) transiently decreased the PWLs 1 h after the treatment, as compared to vehicle-injected rats (Figure 1B and Figure 2A). Intrathecal administration of both BK (21.2 µg) and OLDA (0.42 µg, *n* = 6) together, enhanced this effect, and significantly decreased the PWL for 4 h, with the highest effect 2 h after treatment (Figure 1C and Figure 2B). Furthermore, the PWLs were not affected when selective TRPV1 antagonist SB366791 (0.58 µg) was administered intrathecally, 15 min before BK (21.2 µg) and OLDA (0.42 µg) co-treatment (*n* = 7, Figure 1D). These results showed that enhanced sensitivity to heat induced by co-application of BK and OLDA might be mediated by TRPV1 stimulation.

### 2.2. The Role of TRPV1 in the Mechanical Hypersensitivity Induced by i.t. Bradykinin Treatment

**Figure 3:**
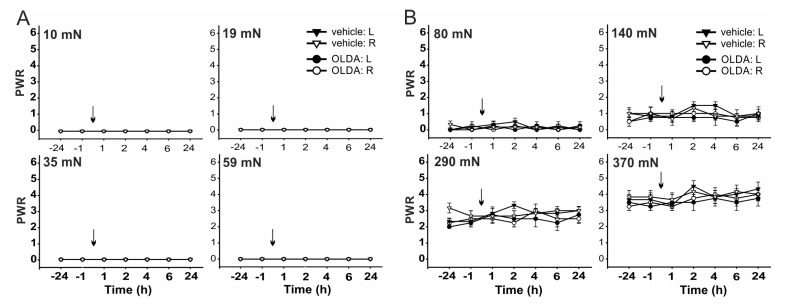
Paw withdrawal responses (PWRs) to mechanical stimulation remained unaffected by intrathecal administration of TRPV1 endogenous agonist OLDA. Administration of OLDA (0.42 µg, *n* = 4) did not change the PWRs evoked by von Frey filaments with bending forces of small intensities ranging from 10 mN to 59 mN (**A**), and stronger intensities ranging from 80 mN to 370 mN (**B**) when compared to vehicle treatment (*n* = 6). Data represent the mean ± S.E.M. of 4 and 6 independent experiments. The time of the i.t. administration is marked by arrows.

Mechanical sensitivity was performed using von Frey filaments with different bending forces. Forces of small intensities 10 mN, 19 mN, 35 mN, and 59 mN did not elicit paw withdrawal response (PWR, Figure 3A) and stronger bending forces 80 mN, 140 mN, 290 mN, and 370 mN evoked PWR in control conditions (Figure 3B). While the PWRs were not changed by vehicle administration, as compared to the control values before injection (*n* = 6). Intrathecal administration of low dose OLDA (0.42 µg, *n* = 4) did not significantly change the mean PWR to the mechanical stimulation with von Frey filaments of any binding force (Figure 3).

**Figure 4:**
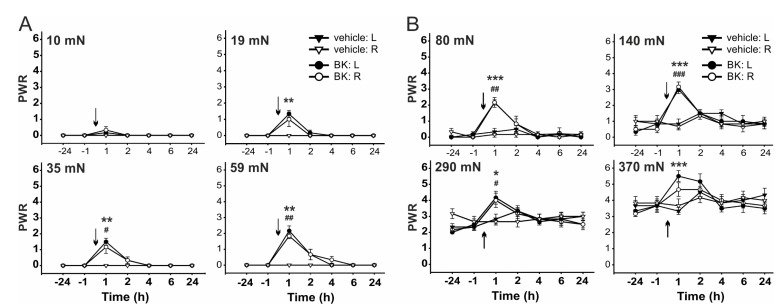
Intrathecal administration of BK evoked mechanical hypersensitivity. Administration of BK (21.2 μg, i.t., *n* = 6) evoked transient mechanical allodynia using von Frey filaments with bending forces 19 mN, 35 mN, and 59 mN (**A**), and hyperalgesia using filaments with bending forces 80 mN, 140 mN, and 290 mN (**B**). Data represent the mean ± S.E.M. of 6 (BK) and 6 (vehicle) independent experiments. Statistical analysis: Mann-Whitney rank test; * *p* < 0.05, ** *p* < 0.01, *** *p* < 0.001 left hindpaw, BK versus vehicle treatment; ^#^ *p* < 0.05, ^##^ *p* < 0.01, ^###^ *p* < 0.001 right hindpaw, BK versus vehicle treatment. The time of the i.t. administration is marked by arrows.

**Figure 5:**
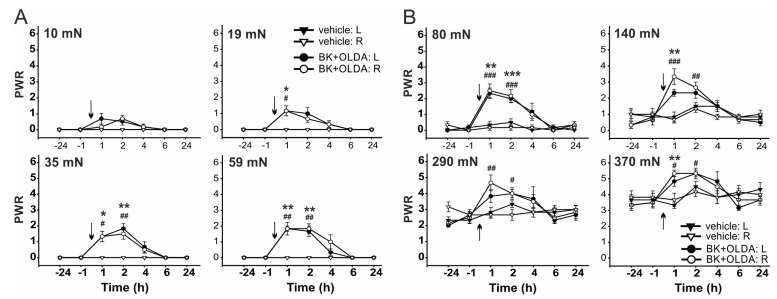
Intrathecal BK administration-induced mechanical hypersensitivity was enhanced by OLDA co-administration. Low dose of OLDA (0.42 µg) and BK (21.2 µg, i.t., *n* = 6) co-administration protracted BK-induced mechanical allodynia using the von Frey filaments, with bending forces of 35 mN and 59 mN (**A**), and hyperalgesia using filaments with bending forces of 80 mN, 140 mN, 290 mN, and 370 mN (**B**). Data represent the mean ± S.E.M. of 6 (BK + OLDA) and 6 (vehicle) independent experiments. Statistical analysis: Mann-Whitney rank test; * *p* < 0.05, ** *p* < 0.01, *** *p* < 0.001 left hindpaw, BK + OLDA versus vehicle treatment; ^#^ *p* < 0.05, ^##^ *p* < 0.01, ^###^ *p* < 0.001 right hindpaw, BK + OLDA versus vehicle treatment. The time of the i.t. administration is marked by arrows.

Administration of BK (21.2 μg, i.t., *n* = 6) transiently increased the PWR 1 h after the treatment, as compared to vehicle-injected rats, when von Frey filaments of bending forces from 19 mN to 370 mN were used (Figure 4 and Figure 7). Transient mechanical allodynia and hyperalgesia were developed. However, i.t. administration of both BK (21.2 μg) and OLDA (0.42 µg, *n* = 6) together increased PWR significantly, at least for 2 h, when the von Frey filaments of bending forces from 35 mN to 370 mN were used (Figure 5 and Figure 7).

**Figure 6:**
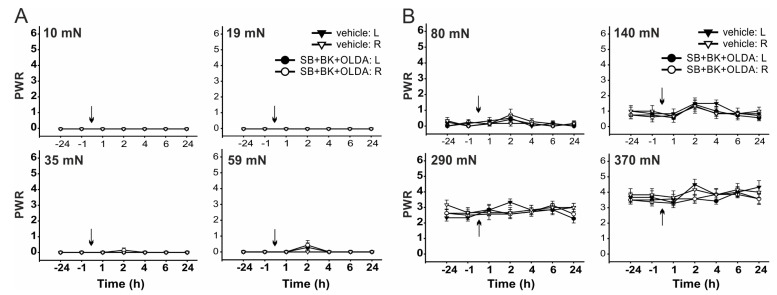
Intrathecal TRPV1 antagonist pretreatment prevented BK- and OLDA-induced mechanical hypersensitivity. Administration of SB366791 (0.58 µg, i.t., *n* = 7), 15 min prior to co-administration of BK and OLDA, completely blocked mechanical allodynia examined with the von Frey filaments, with bending forces ranging from 10 mN to 59 mN (**A**), and hyperalgesia tested with forces from 80 mN to 370 mN (**B**). Data represent the mean ± S.E.M. of 7 (SB + BK + OLDA) and 6 (vehicle) independent experiments. The time of the i.t. administration is marked by arrows.

**Figure 7:**
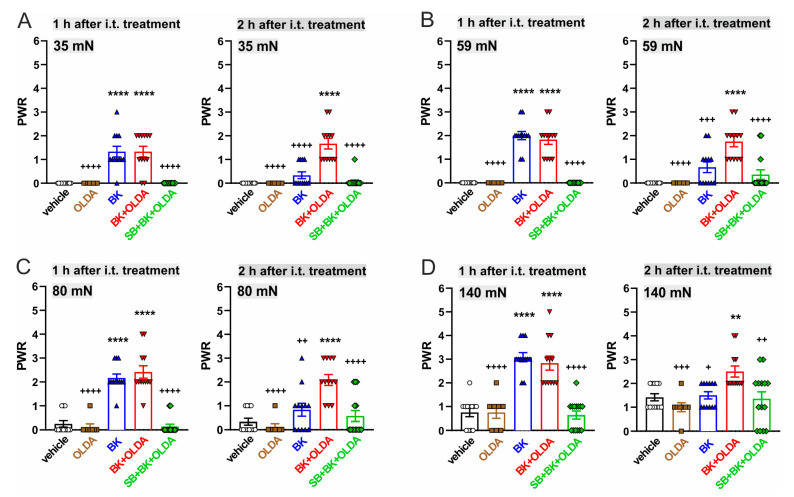
TRPV1 endogenous agonist i.t. treatment enhanced BK-induced mechanical allodynia and hyperalgesia. A detailed comparison of various treatment effects at 1 h and 2 h after the i.t. administration. (**A**,**B**) Co-administration of BK and OLDA evoked a similar allodynia as BK alone, 1 h after i.t. treatment. OLDA enhanced the allodynia 2 h after i.t. treatment. (**C**,**D**) Co-administration of BK and OLDA increased the mechanical hyperalgesia 2 h after i.t. treatment compared to BK alone. Data represent the mean ± S.E.M. with a scatterplot of the individual data points of 4–7 independent experiments. Statistical analysis: One-way ANOVA followed by Tukey’s test; ** *p* < 0.01, **** *p* < 0.0001, versus vehicle i.t. treatment; ^+^ *p* < 0.05, ^++^ *p* < 0.01, ^+++^ *p* < 0.001, ^++++^ *p* < 0.0001, versus BK + OLDA i.t. treatment.

Furthermore, the PWRs remained at control values when the TRPV1 antagonist SB366791 (0.58 µg, i.t.) was administered 15 min before BK (21.2 μg) and OLDA co-treatment (0.42 µg, i.t., *n* = 7, Figure 6 and Figure 7). These results indicate that both mechanical allodynia and hyperalgesia induced by co-application of BK and OLDA might be mediated by TRPV1 stimulation.

## 3. Discussion

Our present study provides evidence that intrathecal administration of OLDA, an endogenous agonist of TRPV1 [31], enhances thermal end mechanical hypersensitivity induced by intrathecal BK treatment. Both thermal and mechanical hypersensitivity evoked by co-administration of BK and OLDA was completely prevented by i.t. pretreatment through selective TRPV1 antagonist SB366791.

In agreement with findings by others [23,24,25], i.t. administration of BK in our experiments induced transient thermal and mechanical hyperalgesia and mechanical allodynia, 1 h after treatment. Administration of low doses of OLDA (0.42 µg) alone did not change the thermal and mechanical sensitivity similarly, as shown in our previous experiments [20]. However, the same concentration of OLDA, co-administered with BK, significantly extended the duration of thermal hyperalgesia to 4 h, and similarly mechanical allodynia and hyperalgesia to 2 h. Importantly, inhibition of spinal TRPV1 through antagonist SB366791, completely prevented all thermal and mechanical hypersensitive states induced by i.t. BK and OLDA treatment. Our results indicate that thermal and mechanical hypersensitivity induced by administration of both BK and OLDA is mediated by activation of spinal TRPV1.

In comparison to BK-induced hypersensitivity, a short-lasting hypoalgesic effect occurring within the first 10 min after BK administration (i.t.) and mediated via activation of bulbospinal inhibitory noradrenergic fibers was demonstrated in awake rats [32]. In our experiments, the rats were treated by short-lasting ether anesthesia, which did not allow examination and detection of very fast effects within minutes after the drug i.t. application. Our behavioral measurements started 1 h after the treatment to avoid any possible residual effect of the anesthesia on paw withdrawal reflexes.

Despite the fact that BK might undergo rapid degradation within seconds [33], we observed a relatively late effect within hours, similar to earlier studies [23,24,25]. This prolonged effect of BK could be underlined by activation of bradykinin receptors signaling resulting in prolonged processes, including other ion channels or receptor activation. In addition, the BK metabolite desArg^9^ BK activates B_1_R, in which the ligand dissociation is slow and the desensitization is limited, in comparison to B_2_R [21]. Both B_1_R and B_2_R belong to the group of G protein-coupled receptors that might undergo unique intracellular signaling from intracellular membranes, which is spatiotemporally distinct from initial signaling of plasmatic membrane [34]. It was demonstrated that B_2_R stimulation-mediated ERK1/2 activation is biphasic, containing an early peak (between 2–5 min), followed by sustained activation for at least 1 h, while βarrestin was involved in this “second wave” of signaling [35]. Moreover, bradykinin regulates glutamatergic synaptic transmission and neuromodulator release in the spinal cord. The bradykinin stimulation of substance P and calcitonin gene-related peptide release from primary nociceptive afferents in the dorsal horn leads to production of prostaglandins, cytokines, and nitric oxide, which could be important for the in vivo effects [21,23].

A crucial role of TRPV1 channels in BK-induced nociceptive responses was suggested in studies on their role in the periphery [26]. Bradykinin acting on B_2_R excited sensory nerve endings in nerve-skin preparation, by activating TRPV1 via the production of 12-lipoxygenase metabolites of the arachidonic acid [36]. Intraplantar injection of BK or B_2_R selective agonist-evoked nociception was mediated by TRPV1, while the phospholipase C pathway activation and lipoxygenase products generation was involved in this process [37]. In contrast, TRPV1-deficient mice maintained the nociceptive response induced by intraplantar injection of high dose of BK, whereas nociceptive behavior was reduced only when a low dose of BK was injected [38]. In comparison, several studies showed a TRPV1 activation-independent mechanism of various responses induced by BK administration to different peripheral sides [39,40,41,42]. TRPV1-dependent and independent mechanisms of BK-induced excitation/nociception were proposed by multiple studies, at the level of peripheral nociceptors. Whereas, two signal transduction pathways arachidonic acid mobilization and PKC activation converged on TRPV1 [43]. Sensitization of TRPV1 by BK through the activation of phospholipase C and downstream PKC that phosphorylates the TRPV1 is well-established, while the level of phosphoinositides could have a role in this process [26,44,45].

At the spinal cord level, both kinin receptors B_1_R and B_2_R contribute to hypersensitive states in vivo [23,24]. It was shown that BK modulates synaptic transmission in the dorsal horn through B_2_R, enhances glutamate release, and increases the sensitivity of ionotropic glutamate receptors. Thus, both presynaptic and postsynaptic mechanisms are involved in the BK-induced effect [23]. Furthermore, low doses of intrathecal B_1_R agonist induced thermal hyperalgesia after subcutaneous pretreatment with capsaicin (24 h), which was blocked by intrathecal administration of TRPV1 antagonist before capsaicin injection. This suggested the contribution of spinal TRPV1 to peripherally injected capsaicin-induced B_1_R upregulation in the microglia [29]. In the spinal cord, it was demonstrated that i.t. BK administration induced activation of PKA, PKC, and downstream extracellular signal-regulated kinase to enhance AMPA and NMDA currents in dorsal horn neurons contributing to hyperalgesia [25]. Previously we showed that BK sensitizes TRPV1 to endogenous agonist OLDA administration in acute spinal cord slices [20]. Our present work showed that spinal TRPV1 receptors are crucial for thermal and mechanical hypersensitivity induced by intrathecal BK administration. Sensitization and indirect activation of TRPV1 at presynaptic terminals of nociceptive primary sensory neurons might be involved in the mechanism. Activation of phospholipase C pathway could sensitize spinal TRPV1 via direct phosphorylation of the channel by PKC, which also mediates the BK-induced lowering of the temperature threshold of TRPV1 activation [46]. In conclusion, our results showed that available endogenous agonists of TRPV1, which might also be generated by activation of spinal BK receptors [47], could play an important role in thermal and mechanical hypersensitivity induced by bradykinin.

## 4. Materials and Methods

### 4.1. Ethics Statement

All experiments were approved by the local Institutional Animal Care and Use Committee of the Institute of Physiology CAS, Prague, Czech Republic (82/2016, 9 September 2016) and were consistent with the guidelines of the International Association for the Study of Pain; the National Institutes of Health Guide for the Care and Use of Laboratory Animals; the U.K. Animals (Scientific Procedures) Act, 1986 and associated guidelines; and the European Communities Council Directive of 24 November 1986 (86/609/EEC). All efforts were made to minimize animal suffering, to reduce the number of animals used, and to utilize alternatives to in vivo techniques, if available.

### 4.2. Animals

Altogether, 29 adult male Wistar rats (200–250 g, Institute of Physiology, CAS, Prague, Czech Republic) were used in this study. The animals were housed in a temperature-controlled facility at 23 ± 2 °C, with free access to food and water, and were maintained on a 12-h light, 12-h dark cycle, and were checked twice a day. All animals were handled only for a necessary period of time, and throughout the experiment, they did not show any signs of stress or illness. Animals were sacrificed at the end of the experiment through deep anesthesia with ketamine (150 mg/kg, i.p., Narkamon, Zentiva Groupe, Prague, Czech Republic) and xylazine (20 mg/kg, i.m., Rometar, Zentiva Groupe, Prague, Czech Republic), with subsequent medulla interruption and exsanguination. No animal was excluded from the study or sacrificed for disease.

### 4.3. Intrathecal Catheter Implantation

Catheters were made of PE-5 tubing. One end of the PE-5 tube was connected to PE-10 tubing using epoxy-glue, and the tubing was filled with sterile physiological saline. For the intrathecal catheter placement, the animals were anesthetized with ketamine (100 mg/kg i.p., Narkamon, Zentiva Groupe, Prague, Czech Republic) and xylazine (10 mg/kg i.m., Rometar, Zentiva Groupe, Prague, Czech Republic). The surgery was performed in a sterile manner. The back of the animal was clipped with an electric razor, a longitudinal incision was made through the skin and the subcutaneous tissue above the spine, and the upper lumbar vertebrae were exposed. The PE-5 end of the catheter was placed into the lumbar subarachnoid space (approximately 0.5 cm in length) and fixed to the spine with dental cement (Duracryl, Spofa, Prague, Czech Republic). The wound was surgically closed in layers, and the PE-10 end of the catheter was exposed on the skin surface of the animal’s back and heat-coagulated. All animals were tested before the control experiments during the behavioral acclimatization period, for signs of any neurological deficits due to the catheter implantation. None of the animals used in the experiments showed any neurological deficits. Animals were left to recover in their cages for at least 5 days. The position of the catheters was verified visually using a dye injection at the end of each experiment.

### 4.4. Behavioral Tests Procedures

Responsiveness to mechanical stimulation was tested with the von Frey (VF) filaments. Each VF monofilament was calibrated on a top-loading electronic balance and the force needed to bend the filament was measured. The calibration of the filaments was re-checked both before and at the end of each experiment, to ensure that the stimulus intensity remained unchanged. Rats were placed on an elevated plastic mesh (0.5 × 0.5 cm perforations) under a nonbinding, clear plastic cage, and were left to adapt to the testing environment for at least 15 min. VF filaments with bending forces of 10, 19, 35, 59, 80, 140, 290, and 370 mN were used to deliver punctuate mechanical stimuli of varying intensity to the plantar aspect of each hindpaw, from below the mesh floor. The use of the von Frey filaments of different strengths enabled us to distinguish between mechanical allodynia, using filaments that did not evoke responses before the treatment (10–59 mN), and mechanical hyperalgesia, using filaments that in contrast did evoke PWR before the treatment (80–370 mN), similar to our previous studies [4,5]. Each stimulus was applied 6 times, each poke spaced 2 s apart, and sequential monofilaments were applied in ascending order of stiffness. Care was taken to stimulate certain location on the plantar surface (the area of the heel) on both hind paws. The number of withdrawal responses to the VF filament stimulation was recorded. Shifts in weight or voluntary movements associated with locomotion were not counted as a withdrawal response. Baseline responses were determined in all animals before the experimental procedures.

Subsequent to the von Frey test (5 min), the responsiveness to thermal stimulation was tested with radiant heat applied to the plantar surface of each hind paw. Rats were placed under a nonbinding, clear plastic cage on a clear 3 mm thick glass plate, elevated to allow maneuvering of a controlled, radiant heat source underneath. Each rat was left to adapt to the testing environment for at least 15 min, prior to any stimulation. A focused light source with halogen bulb was used to deliver the heat stimuli (50 W, Dittel, Prague, Czech Republic). The radiant heat was applied to the plantar surface of the hind paw, to the area of the heel, and to the same location on both hind paws. The paw withdrawal latencies (PWLs) were measured with a digital timer. A 30 s cutoff time was imposed on the stimulus duration to prevent tissue damage. PWLs were tested 3 times in each hindpaw with at least 5 min between trials. Baseline PWLs were determined in all animals before any experimental procedure. The experimenter was blinded to the type of treatment in all behavioral tests.

### 4.5. Drugs

A total of 50 μM N-Oleoyldopamine (OLDA, Tocris Bioscience, Bristol, UK) was prepared from 50 mM stock solution (OLDA dissolved in DMSO). This 50 mM OLDA stock solution was further dissolved in 0.9% NaCl to reach 50 μM concentration. A total of 1 mM BK (bradykinin acetate, Sigma-Aldrich, Prague, Czech Republic) was prepared from 16 mM stock solution and 100 μM SB366791 (Tocris Bioscience, Bristol, UK) was made of 100 mM stock solution (SB366791 dissolved in DMSO). The stock solution was further dissolved in 0.9% NaCl solution to reach 100 μM concentration.

On the day of the experiment, the drugs were injected intrathecally under short-lasting ether anesthesia. After the injection, the animals were left to recover in their cages before the behavioral testing procedure started.

### 4.6. Experimental Groups

All rats were tested for baseline responses to mechanical and thermal stimuli first, 24 h and 1 h before any drug application. Then, in the first group of animals, 0.42 µg of OLDA in the volume of 20 μL (50 μM, *n* = 4) was injected into the catheter, followed by 50 μL of physiological saline. One hour later, the behavioral testing for responsiveness to mechanical and thermal stimulation was performed. The responses were then tested again 2, 4, 6, and 24 h later. In the second group of animals, 21.2 μg of BK in the volume of 20 μL (1 mM, *n* = 6) was injected intrathecally, followed by 50 μL of physiological saline. Then, these animals underwent the same behavioral testing procedure as the previously described experimental group.

In the third group of animals, on the first day of the experiment, 21.2 μg of BK in the volume of 10 μL (2 mM) and 0.42 µg of OLDA in the volume of 10 μL (100 μM, *n* = 6) was applied into the catheter, followed by 50 μL of physiological saline. The animals were further tested for responsiveness to mechanical and thermal stimuli, according to the same testing protocol as described above. In the fourth group of animals, TRPV1 antagonist SB366791 (0.58 μg, 20 μL, *n* = 7) was first administered intrathecally. Fifteen min after SB366791 administration, 21.2 μg of BK (10 μL) and 0.42 µg of OLDA (10 μL) was applied into the catheter, followed by 50 μL of physiological saline. These animals underwent identical behavioral testing, as described above.

### 4.7. Data Analysis

The withdrawal responses evoked during the mechanical stimulation with the von Frey filaments were evaluated as present (1) or absent (0), and a mean value from the 6 trials for each filament strength was calculated. The mean values from all rats in the group were then averaged and means ± S.E.M. were calculated. Paw withdrawal latencies evoked by heat stimuli were averaged from the 3 trials for each hindpaw and mean ± S.E.M. were calculated for each experimental situation and time-point. For the statistical analyses, the Mann-Whitney rank test and One-way ANOVA followed by Tukey’s test were used.

## Data Availability

All relevant data are within the paper.
